# Genomic Characterization of Piscicolin CM22 Produced by *Carnobacterium maltaromaticum* CM22 Strain Isolated from Salmon (*Salmo salar*)

**DOI:** 10.1007/s12602-024-10316-1

**Published:** 2024-07-03

**Authors:** Elías González-Gragera, J. David García-López, Claudia Teso-Pérez, Irene Jiménez-Hernández, Juan Manuel Peralta-Sánchez, Eva Valdivia, Manuel Montalban-Lopez, Antonio M. Martín-Platero, Alberto Baños, Manuel Martínez-Bueno

**Affiliations:** 1 Department of Microbiology, University of Granada, Avda. Fuentenueva, S/N, 18071 Granada, Spain; 2 Department of Zoology, University of Seville, Avda. Reina Mercedes, 6, 41012 Seville, Spain; 3 Institute of Biotechnology, University of Granada, 18071 Granada, Spain

**Keywords:** *Carnobacterium maltaromaticum*, Antimicrobials, Bacteriocins, Probiotics, Piscicolin, Food pathogens

## Abstract

*Carnobacterium maltaromaticum* is a species of lactic acid bacteria (LAB) that has been isolated from various natural environments. It is well-known for producing a diverse spectrum of bacteriocins with potential biotechnological applications. In the present study, a new psychrotolerant strain of *C. maltaromaticum* CM22 is reported, isolated from a salmon gut sample and producing a variant of the bacteriocin piscicolin 126 that has been named piscicolin CM22. After identification by 16S rRNA gene, this strain has been genomically characterized by sequencing and assembling its complete genome. Moreover, its bacteriocin was purified and characterized. In vitro tests demonstrated that both the strain and its bacteriocin possess antimicrobial activity against several Gram-positive bacteria of interest in human and animal health, such as *Listeria monocytogenes*, *Clostridium perfringens*, or *Enterococcus faecalis*. However, this bacteriocin did not produce any antimicrobial effect on Gram-negative species. The study of its genome showed the genetic structure of the gene cluster that encodes the bacteriocin, showing a high degree of homology to the gene cluster of piscicolin 126 described in other *C. maltaromaticum*. Although more studies are necessary concerning its functional properties, this new psychrotolerant strain *C. maltaromaticum* CM22 and its bacteriocin could be considered an interesting candidate with potential application in agri-food industry.

**Supplementary Information:**

The online version contains supplementary material available at 10.1007/s12602-024-10316-1.

## Introduction

In recent years, European consumers have changed their consumption habits and dietary models towards natural foods, locally produced foods, less industrially processed foods, and foods free of synthetic additives [1]. In addition, there is a growing concern about sustainable animal production and consumption of antibiotic-free animal products [2, 3]. The use of probiotics or their bacteriocins could be a good alternative to the use of synthetic compounds for the livestock and food industry, increasing animal welfare and food safety [4].

Bacteriocins represent a promising alternative to the use of antibiotics or chemical additives; however, their commercial use has not yet expanded significantly, mainly due to regulatory aspects [5]. Bacteriocins usually alter the membrane integrity of susceptible cells [6] and cause the leakage of intracellular solutes, ultimately leading to cell death. Furthermore, bacteriocins usually have a narrow inhibition spectrum that targets genera or species closely related to the producing strain [7]. These compounds are small ribosomally synthesized peptides typically produced by Gram-positive bacteria. They primarily inhibit the growth or cause the demise of other Gram-positive bacteria [8].

Bacteriocins of lactic acid bacteria (LAB) are the most studied ones due to the GRAS (generally recognized as safe) status that most of these species have. Thus, their bacteriocins can be safely used as natural preservatives in food [9]. However, nisin is currently the only bacteriocin that can be used as an authorized additive. Nevertheless, pediocin is being considered for its forthcoming commercial application in food preservation purposes, and its use is safeguarded by several patents [10, 11]. Therefore, there is a big niche for new broad-spectrum molecules in this market [12]. These molecules are of great interest in the agro-food industry, since they can be more easily authorized compared to other synthetically produced chemical compounds. Their aim is to enhance food safety, improve the quality, and extend the shelf life of various food products.

*Carnobacterium* is a genus of LAB which produces several bacteriocins belonging to classes IIa, IIc, and cyclic bacteriocins such as carnobacteriocin BM1, carnobacteriocin B2, carnobacteriocin A, carnocyclin A, piscicolin 126, and piscicocin CS526 [13]. The recent characterization of these bacteriocins has revealed interesting activity against several food-borne microorganisms, such as *Enterococcus faecalis* or *Listeria monocytogenes* [14]. This suggests that studying them could provide novel biopreservation alternatives. Finally, different strains of LAB, including *Carnobacterium* species with probiosis properties [15–17], have been described. The use of these bacteriocinogenic strains is an interesting tool for the agri-food sector. For instance, some LAB have shown an increase in productive parameters in farm animals [18, 19], an improvement in animal welfare by reducing infections [20, 21], and an increase in food safety from the source to the consumer [22, 23].

The aim of this work to explore the antibiosis properties of *Carnobacterium maltaromaticum* CM22, a bacteriocinogenic strain isolated from salmon. For this purpose, we have sequenced its genome, characterized this strain, and purified and identified its bacteriocins.

## Material and Methods

### Bacterial Strains and Culture Conditions

Different bacterial strains were used to test antimicrobial activity of *C. maltaromaticum* CM22, or as positive/negative controls for the specific characteristics investigated (Table 1). The microorganisms used in this study were obtained from the CECT (Spanish Collection of Type Cultures) and wild isolates from our collection (Table 1). All strains were routinely cultivated on either trypticase soy broth (TSB, Scharlau, Barcelona, Spain) at 37 °C and stored, or 4 °C on the respective agar slants.

**Table 1:** Bacterial strains employed in the characterization of *Carnobacterium maltaromaticum* CM22. CECT, Colección Española de Cultivos Tipo (Spanish collection of type strains); BIO160, research group from University of Granada

**In vitro antibiosis characterization**	**Source**
*Listeria innocua* CECT 4030	Fresh Cheese
*Enterococcus faecalis* JH2-2 [24]	Clinical isolate
**Antimicrobial test**	
*Listeria monocytogenes* CECT 4032	Clinical isolate of meningitis
*Listeria monocytogenes* CECT 5366	Clinical isolate
*Clostridium perfringens* CECT 486	Boiled salt beef
*Clostridium perfringens* CECT 821	Sheep
*Enterococcus faecalis* S-47 (Gálvez et al., 1989)	Clinical isolate
*Lactobacillus fermentum* DMC-015 (BIO160 collection)	Spoiled sauce
*Leuconostoc mesenteroides* DMC-07 (BIO160 collection)	Goat milk
*Leifsonia aquatica* CECT 535	Water
*Streptococcus phocae* subsp*. salmonis* CECT 7921	Atlantic salmon
*Staphylococcus aureus* CECT 976	Ham involved in food poisoning incident
*Escherichia coli* CECT 516	Human feces
*Salmonella enterica* CECT 7159	Egg
*Yersinia enterocolitica* O9 CECT 754	Human feces

### Screening and Isolation Procedure

The strain *C. maltaromaticum* CM22 was isolated during a LAB biodiversity study from a fish farm in Puerto Montt (Chile). A salmon (*Salmo salar*) that weighed 2 kg was hermetically packed in pre-sterile polyethylene bag. The fish was washed with sterile distilled water and then, the surface was cleaned with 70% alcohol. The fish gut was removed under aseptic conditions and washed with 0.85% saline solution. The gut sample was placed in a sterile bag for homogenization in a digester (Stomacher, VWR International, Radnor, Pennsylvania, USA) with sterile buffered peptone water (Scharlau). The digestion product was plated on MRS agar (Scharlau) and incubated anaerobically at 5 °C, 10 °C, and 30 °C for 2–7 days. Colonies were then randomly selected, replicated on MRS agar plates (Scharlau), and incubated for 72 h. Screening was carried out on 180 psychrotolerant strains searching for antimicrobial activity. This activity was examined for antibiosis production by overlaying the strains with 6 mL of soft agar inoculated with 2% stationary-phase cultures of *E. faecalis* JH2-2 and *L. innocua* CECT 4030 (Table 1). After incubation at 30 °C, strains that show antibiosis would inhibit the growth of both indicator strains, showing halos around its colony. In this sense, *C. maltaromaticum* CM22 strain was selected for further analysis due to its highest antibacterial activity against both strains.

### Spectrum of Antibiosis

The antimicrobial activity of *C. maltaromaticum* CM22 strain was studied against some of the most common human and animal pathogens and food spoilage bacteria (both Gram-negative and Gram-positive species) (Table 1) by the agar diffusion test described by Alonso et al. [25]. Target bacteria were grown in Brain Heart Infusion (Scharlau), *C. maltaromaticum* CM22 strain was grown in MRS (Scharlau), and both cultures were incubated overnight at 30 °C. Target bacteria were spread in Brain Heart Infusion Agar (BHA) Petri dishes using a bacterial suspension adjusted to form a bacterial lawn, and once the plate was dried, drops (15 µL) of a stationary-phase culture of the *C. maltaromaticum* CM22 strain were dispensed onto the plates. After incubation at 30 °C for 24 h, the plates were examined for the absence/presence of inhibition zones and the results were interpreted as positive ( +) or negative ( −). Tests against each indicator strain were performed in duplicate.

In addition, a well-diffusion technique [26] was carried out with the aim of detecting antibiosis in liquid medium. Therefore, sterile stainless-steel towers 8 mm in diameter × 10 mm in height (Oxford Towers, Scharlab, Barcelona, Spain) were deposited on plates with a base layer of tryptic soy agar (Scharlau). An overlay of tryptic soy agar (0.8% agar) was then melted, inoculated with the target strains, and poured evenly onto the plate. After solidification of the overcoating layer, the cylinders were removed. Then, holes were filled individually with 100 μL of *C. maltaromaticum* CM22 culture supernatant previously filtered through a 0.22-µm polyethersulfone (PES) syringe-driven filters (Merck Millipore, Carrigtwohill, Ireland). Finally, the plates were incubated aerobically at 37 °C for 24–48 h. After incubation, the clear zones were measured using a calliper. Tests against each indicator strain were performed in duplicate.

### *Carnobacterium maltaromaticum* CM22 Strain Identification and Genomic Characterization

Preliminary identification was based upon phenotypic characteristics, including cell morphology and Gram staining, catalase activity, API50 system (BioMérieux, Craponne, France), and ability to grow at 10 and 45 °C and in the presence of 6.5% (w/v) NaCl.

For the genotypic characterization, genomic DNA was extracted from a pure liquid culture according to Martín-Platero et al. [27]. This genomic DNA was used as a template for 16S rDNA amplification using the WO12 and WO1 primers according to Ogier et al. [28]. PCR reactions were performed in a total volume of 50 µL containing 5 µL of 10X Taq reaction buffer, 1.5 mM of MgCl_2_, 400 μM of dNTPs, 0.4 μM of the primers WO1 and WO12, 1 U of Taq DNA polymerase (IBIAN Technologies, Zaragoza, Spain), and 1 µL (20–50 ng) of template DNA. The amplification program consisted of an initial denaturing step at 94 °C for 4 min followed by 30 cycles at 94 °C for 30 s, 50 °C for 30 s, and 72 °C for 60 s and a final extension of 72 °C for 2 min. A 700-bp fragment of the 16S rDNA gene containing the V1–V4 variable regions was obtained, purified with a Perfectprep Gel Cleanup kit (Eppendorf, Hamburg, Germany).

The genome was sequenced with the Illumina HiSeq4000 platform by STAB VIDA (Caparica, Portugal). Sequencing libraries were constructed with an insert size of 300 bp, sequencing 150 bp from each end. The assembly of the readings was carried out with SPAdes 3.13.0 [29]. The scaffold was made with MeDuSa 1.6 [30]. GapFiller 1.10 was used to close the scaffold gaps [31]. In addition, the annotation was made with Prokka 1.13.3 [32]. To perform the comparative genomic analysis, Artemis [33] and BLAST [34] were used. Finally, to determine if the *C. maltaromaticum* CM22 genome was associated with the structural gene of any bacteriocin, a TBLASTN (version 2.10.1 +) was run with a 10^−6^ *e*-value threshold [34, 35] between our genomes and bacteriocins from the BACTIBASE databases [36].

### Bacteriocin Production, Purification, and Identification

A flask containing 1 L of Brain Heart Infusion (BHI, Scharlab) adjusted to pH 6.5 was inoculated at 2% with a stationary-phase culture of *C. maltaromaticum* CM22 strain. The cultured flask (1 L) was incubated at 28 °C overnight and centrifuged for 20 min at 4 °C and 3724 rpf (Beckman Coulter, California, USA), collecting the supernatant that was tested by the well-diffusion technique [26].

The bacteriocin present in the medium was recovered by Carboxymethyl-Sephadex CM-25 (Merck Life Science S.L.U. Madrid, Spain) cation exchange chromatography. The recovery of the bacteriocins was carried out using the method described by Abriouel et al. [37] with some modifications. The supernatants at pH 7.0 were mixed with 1 N NaOH, 200 mL of Carboxymethyl Sephadex CM-25 (GE Healthcare, Madrid, Spain) and left stirring for 30 min. Afterwards, the supernatants were removed, and the CM-25 gel was transferred to a cylindrical filtering funnel with a plate porosity of 100–160 microns (Pobel, Madrid, Spain). The gel was washed with 3 volumes of distilled water, followed by 3 volumes of 1 M NaCl and 3 volumes of 1.5 M to elute the adsorbed bacteriocin. During the process, 50-mL fractions were collected and filtered (0.22 μm PES, Merck Millipore, Cork, Ireland), and its activity was measured using the afore mentioned well-diffusion technique.

The fractions that showed antibacterial activity from cation exchange chromatography were repurified by reversed-phase extraction on a C-18 solid support. For this, 5 g of C-18 resin (Waters) was used in a plastic column (2.5 × 10 cm). The C-18 column was washed with 10 mL of isopropanol to acetonitrile 2:1 added with 0.1% trifluoroacetic acid (solvent B) for each gram of resin for equilibration. Subsequently, it was washed with 25 mL of 0.1% trifluoroacetic acid in MilliQ water (solvent A). The active fractions were passed through the column and another wash was carried out with 15 mL of solvent A. Finally, the samples were eluted with increasing concentration of solvent B. Thus, 15 mL 30% solvent B, 15 mL 60% solvent B, and 15 mL solvent B were collected in separate tubes and tested for antimicrobial activity [26]. Those fractions that showed antimicrobial activity were lyophilized and resuspended in solvent A for repurification by reversed-phase high-performance liquid chromatography (RP-HPLC). Purification was performed using a Zorbax Eclipse XBD C18 5 µm particle 4.6 × 150 mm column (Agilent) and an Agilent 1100 analytical HPLC. The machine was operated at 1 mL/min flow rate using a protocol consisting of an equilibration step of 5 min at 5% solvent B, 1 min 5–40% solvent B, 5 min 40% solvent B, and a separation step of 40–95% solvent B gradient for 20 min.

Finally, the peptide nature of the purified bacteriocin was determined by proteolysis with pancreatic trypsin (10 mg/mL in 50 mM Tris–HCl, pH 7.2) (Merck) using a 1:1 enzyme to substrate (v:v) ratio at 37 °C for 2 h. After enzymatic treatment, loss of activity was monitored by well-diffusion against *L. innocua*.

Nisin was purified from commercial Nisaplin as indicated by Slootweg and coworkers [38] rendering HPLC-grade nisin.

### Determination of the Minimal Inhibitory Concentration of Nisin and Piscicolin CM22 in Solid Media

HPLC-purified nisin and piscicolin CM22 were solubilized in 0.05% acetic acid, and their concentrations were measured using the Bio-Rad protein assay. Both peptides were adjusted to a final concentration of 0.6 g/L. Twofold serial dilutions of each peptide were done. Subsequently, 5 µL of each dilution was spotted onto a 7-mL overlay of soft BHI 0.7% agar inoculated with 100 µL of a stationary-phase culture of the susceptible strain *L. monocytogenes* CECT4032. The minimal inhibitory concentration was determined as the last concentration at which clear inhibition was observed.

### Determination of the Molecular Weight and MALDI-TOF Identification

The molecular weight of the purified bacteriocin was determined by MALDI-TOF. For this purpose, a tricine sodium dodecyl sulfate–polyacrylamide gel electrophoresis (SDS-PAGE) (Sigma-Aldrich, Madrid, Spain) system was used. Electrophoresis was carried out using the Criterion™ Cell (300 V, 20 min). The gel was washed with sterile distilled water and fragments were fixed with 25% (v/v) isopropanol and 10% (v/v) glacial acetic acid (Sigma). One of the fixed gels was stained with Coomassie blue (Sigma) overnight with constant shaking, and then, a solution of water and methanol was used to attenuate the gel. Bands were revealed by a gel documentation system. For the antimicrobial activity test, the other gel fragment without fixation process was washed [39], placed in a sterile Petri dish, and then covered with Brain Heart Infusion agar containing the indicator strain *L. innocua CECT4030*. Finally, the sample of fixed gel was stored in sterile distilled H_2_O for identification by matrix-assisted laser desorption ionization-time of flight mass spectrometry (MALDI-TOF MS) using a Voyager-DE PRO spectrometer from Applied Biosystems.

## Results

### Isolation and Identification of *Carnobacterium maltaromaticum* CM22 Strain

The *C. maltaromaticum* CM22 strain was selected during a study on bacteriocin production among LABs isolated from a Chilean salmon fish farm. This psychrotolerant strain, capable of growing within a temperature range of 5 to 30 °C, exhibited a notable inhibitory spectrum against both target bacteria, *E. faecalis* JH2-2 and *L. innocua* CECT 4030. Initially, this strain was identified as *C. maltaromaticum* based on the API50 system. Afterward, a similarity analysis of the 16S rDNA sequence obtained by PCR amplification confirmed the *C. maltaromaticum* species identity with a similarity of 925/928 bp (99.7%) compared to the reference sequence M58825 of *C. maltaromaticum* DSM 20342.

### Assessment of Antimicrobial Potential

The antibacterial effect was assayed using agar diffusion tests, and *C. maltaromaticum* CM22 strain was found to inhibit the growth of 9 out of the 13 tested species (Table 2). The target strains included representative members of food-spoilage bacteria and potential pathogens for animals and humans. *C. maltaromaticum* CM22 showed antibacterial activity both in solid and in liquid media against all the Gram-positive bacteria tested, except against *S. aureus*. The antibacterial activity demonstrated against *Listeria* strains, which presented inhibitory zones of up to 33 mm in diameter using the well-diffusion method, was noteworthy (Supplementary Fig. 1S; Table 2). *C. maltaromaticum* CM22 did not show antimicrobial activity against any of the Gram-negative bacteria tested. These results suggest the ability of *C. maltaromaticum* CM22 to produce specific extracellular agents against other Gram-positive strains.

**Table 2:** Inhibition produced by *Carnobacterium maltaromaticum* in antibiosis assays (drops of *C. maltaromaticum* on solid medium) and antimicrobial activity (100 µL of *C. maltaromaticum* CM22 supernatants in the well-diffusion method). One arbitrary unit (AU) was defined as the highest dilution that resulted in a clearly visible area of inhibited growth on a bacterial-seeded lawn in a well. The titer of activity in arbitrary units per milliliter was determined by taking the reciprocal of this dilution

**Strain used for antimicrobial test**	**Antimicrobial activity (mm)**	**Activity AU/mL**
*Listeria monocytogenes* CECT 4032	32 ± 1	640 AU/mL
*Listeria monocytogenes* CECT 5366	33 ± 1	640 AU/mL
*Clostridium perfringens* CECT 486	19 ± 1	160 AU/mL
*Clostridium perfringens* CECT 821	18 ± 1	160 AU/mL
*Enterococcus faecalis* S-47	23 ± 1	320 AU/mL
*Lactobacillus fermentum* CECT 4676	19 ± 1	160 AU/mL
*Leuconostoc mesenteroides* DMC-07	23 ± 0.5	320 AU/mL
*Leifsonia aquatica* CECT 535	18 ± 1	160 AU/mL
*Streptococcus phocae* subsp. *salmonis* CECT 7921	17 ± 1	160 AU/mL
*Staphylococcus aureus* CECT 976	0	Absence
*Escherichia coli* CECT 516	0	Absence
*Salmonella enterica* CECT 7159	0	Absence
*Yersinia enterocolitica* O9 CECT 754	0	Absence

In addition, to establish an initial approximation of the potency of *C. maltaromaticum* CM22, a comparison was conducted against other described bacteriocins producers known for their potent antilisterial activity (Supplementary Fig. 2S). Arbitrary units per milliliter (AU/mL) were quantified against nisin and pediocin from *Lactococcus lactis* and *Pediococcus acidilactici*, respectively (Sigma-Aldrich), using *L. monocytogenes* CECT 4032 as the target strain. The results showed 640 AU/mL for nisin and 320 AU/mL for pediocin. This suggests, pending further investigation into its spectrum of action, that antimicrobial potency of *C. maltaromaticum* CM22 could be comparable to that of nisin and slightly superior to that of pediocin.

### Purification and Characterization of Antimicrobial Agents

After purification using cation exchange chromatography, both the NaCl eluates and the initial supernatant showed antimicrobial activity, with inhibition halos ranging between 15 and 20 mm in diameter against *L. innocua* CECT 4030. Samples that showed activity after chromatography with CM-25 were then subsequently pooled and further purified by reversed-phase chromatography with C-18. The different eluted fractions were assayed, showing inhibition halos of 19–20 mm in diameter against both *E. faecalis* and *L. innocua*, respectively.

The C18 fractions with the highest antimicrobial activity were lyophilized, resuspended in solvent A, then mixed, and HPLC purified. The different peaks in the chromatogram were collected separately, and only the peak with 18-min retention time showed antimicrobial activity and was further characterized (Supplementary Fig. 3S).

The molecular mass of the different eluted fractions from C-18 was estimated by polyacrylamide gel electrophoresis under denaturing conditions (tricine SDS-PAGE) and subsequent antimicrobial activity. Only those major fractions with antimicrobial activity were analyzed. Polyacrylamide gel electrophoresis showed a single band corresponding to a substance with a molecular weight lower than 6 kDa (Supplementary Fig. 4S). Furthermore, the antimicrobial susceptibility test, using the overcoat technique with *L. innocua* CECT 4030 as an indicator strain corroborated the presence of an inhibitory substance of peptide nature,with a low molecular weight.

**Fig. 1:**
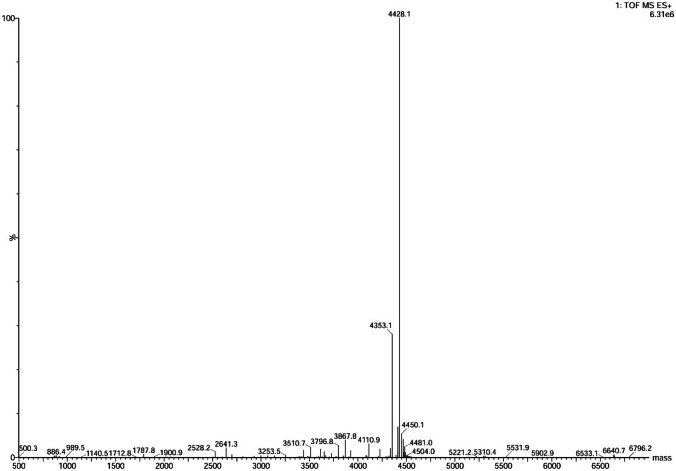
Analysis by MALDI-TOF–MS of the 18-min fraction of the strain *Carnobacterium maltaromaticum* CM22 purified by RP-HPLC

The determination of the molecular mass of the active fractions obtained by RP-HPLC (at 18 min) was performed by MALDI-TOF mass spectrometry. A well-defined peak with a mass of 4428.1 Da (Fig. 1) was observed, which presents a great similarity with literature data piscicolin 126 (4416.6 Da). Finally, in order to confirm the nature of the molecule, the treatment of the filtered supernatant with a protease (pancreatic trypsin, Merck) in proportion (1:1) (v:v) resulted in the total loss of antimicrobial activity (data not shown).

The purified fraction maintains the activity against the sensor strains included in Table 2. Furthermore,the potency of purified piscicolin CM22 was assayed against *L. monocytogenes* and compared to that of the reference bacteriocin nisin. For this,  both compounds were solubilized at the same concentration and serial dilutions were spotted onto the susceptible strain. *L. monocytogenes* was inhibited by both compounds, with piscicolin CM22 remaining active up to 4.7 mg/L and 2.3 mg/L against the strains CECT4032 and CECT5366, respectively, and nisin at 18.7 mg/L under these conditions. Additionally, we conducted the same test on the intermediate susceptible strain *L. aquatica* obtaining a minimal inhibitory concentration of 300 mg/L and 37,5 mg/L of piscicolin CM22 and nisin, respectively. These data are consistent with the higher potency of type IIa bacteriocins against *Listeria* strains as reported in literature.

### Genomic Analysis of *Carnobacterium maltaromaticum* CM22

After sequencing, assembly, and annotation, *C. maltaromaticum* CM22 genome showed a size of 4.08 Mb, with a GC content of 33.59%. Additionally, 3797 protein-coding DNA sequences (CDS), 62 tRNA genes, and 10 rRNA were found in its genome.

**Fig. 2:**
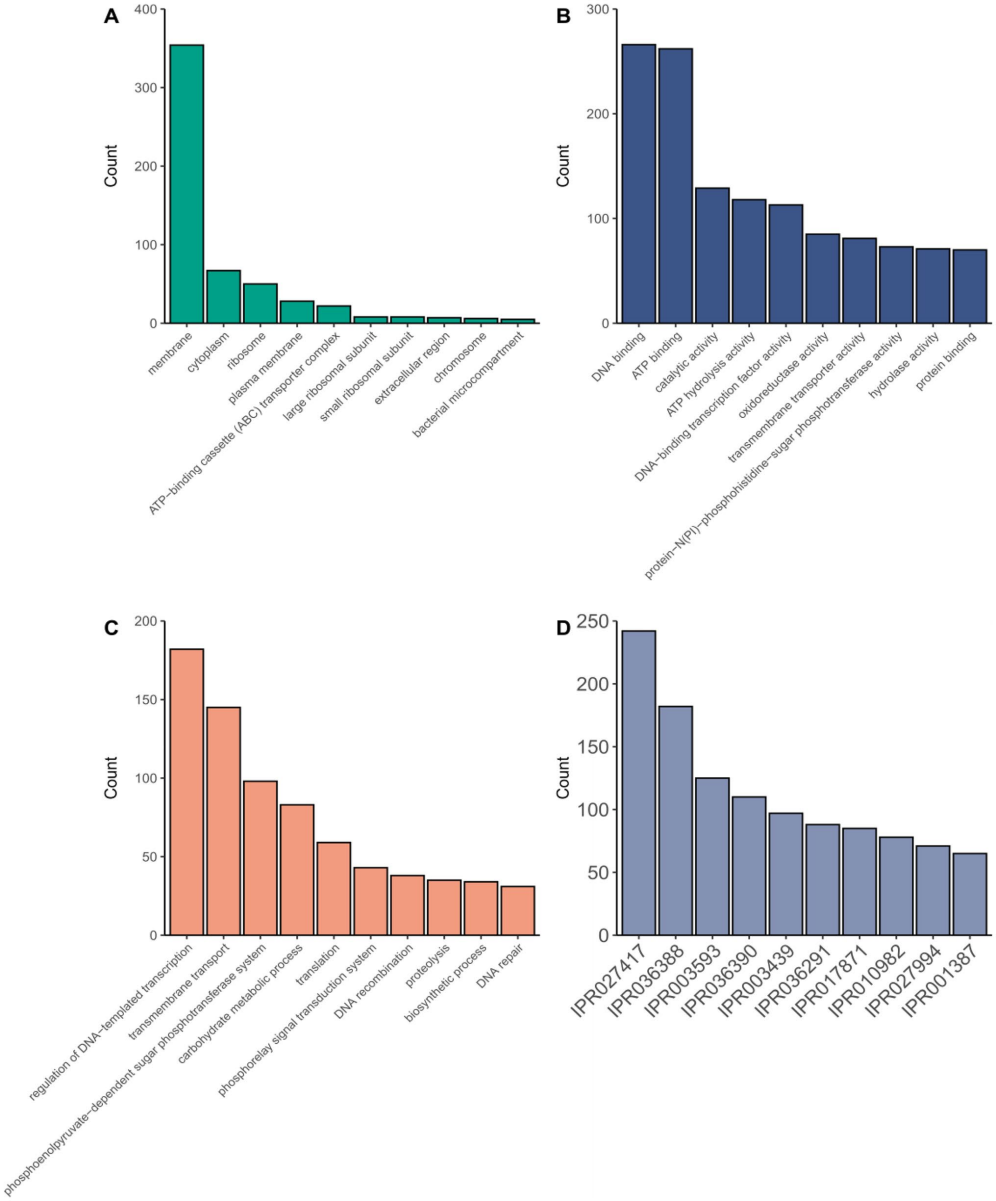
Functional annotation and analysis of the *Carnobacterium maltaromaticum* CM22 genome. Top ten most abundant Gene Ontology (GO) term counts for **A** cellular components, **B** molecular function, **C** biological processes categories, and **D** InterProScan for gene families

Gene Ontology (GO) analysis of the protein-coding genes assigned 5585 GO terms to 2031 (61%) genes. Of the total GO terms, 605 (10.8%), 3209 (57.5%), and 1771 (31.7%) were assigned to cellular components, molecular functions, and biological processes respectively. Membrane components, DNA binding, and regulation of DNA-template transcription were the most abundant terms among each category (Fig. 2A–C). InterProScan detected 3329 proteins from *C. maltaromaticum* CM22 genome. A total of 10,610 families were assigned to 3125 proteins (93.9%, 3125/3329), where P-loop containing nucleoside triphosphate hydrolase family (IPR027417) obtained the highest assignation, followed by winged helix-like DNA-binding domain superfamily (IPR036388) and the AAA + ATPase domain (IPR003593) (Fig. 2D).

**Fig. 3:**
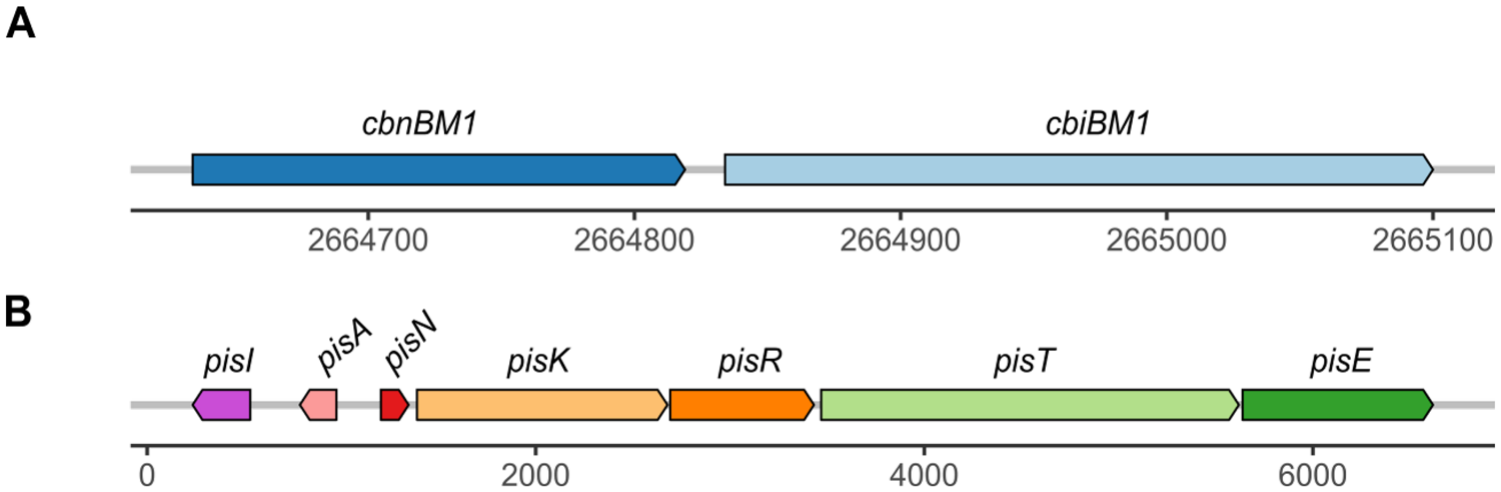
Genetic organization of carnobacteriocin BM1 and piscicolin CM22 bacteriocins. **A** Two identified open reading frames (ORFs) are depicted in the carnobacteriocin BM1 gene cluster: *cbnBM1* (dark blue arrow) corresponds to the structural gene while *cbiBM1* (light blue) encodes an immunity protein. **B** Gene cluster of piscicolin 126. A total of 8 ORFs are depicted: *pisI* (violet arrow) is the putative accessory protein; *pisA* (pink arrow) is the structural gene for the bacteriocin; *pisN* (red arrow) codes for a putative induction peptide; *pisK* and *pisR* (light and dark orange arrows) encode an histidine kinase and a response regulator respectively; *pisT* (light green arrow) is the ABC transporter, and *pisE* (dark green arrow) encodes a putative transport accessory protein

Results of TBLASTN against the BACTIBASE database showed that genes for piscicolin 126 variant, namely piscicolin CM22, and carnobacteriocin BM1 bacteriocins were present in *C. maltaromaticum* CM22 genome. The class IIa bacteriocin carnobacteriocin BM1 (6572 Da) gene cluster was formed by two open reading frames (ORFs), *cbnBM1* and *cbiBM1* (Fig. 3A). *cbnBM1* corresponded to the structural gene and would be responsible for the production of a protein with 61 amino acid residues, while *cbiBM1* encoded an immunity protein [14, 40–42].

Piscicolin CM22 gene cluster contained at least eight ORFs (Fig. 3B). The structural gene (*pisA*) encoded a protein of 62 amino acid residues of which the first 21 corresponded to a signal peptide. *pisN* encoded a putative induction peptide (IP) that might be involved in regulating piscicolin 126 production. *pisK* and *pisR* were part of a two-component system where *pisK* encoded a histidine kinase and *pisR* encoded for a response regulator. *pisT* encoded for the bacteriocin ABC transporter, *pisE* encoded for the putative transport accessory protein, and *pisI* encoded for the immunity protein. Despite the presence of the genetic sequence responsible for the synthesis of carnobacteriocin BM1 in its genome, MALDI-TOF analysis of the active fraction did not detect the presence of this bacteriocin. This fact may be attributed to the absence of BM1 production or its exclusive synthesis in specific circumstances that are unknown at the moment, such as the different temperature conditions [43].

The genomic analysis of *C. maltaromaticum* CM22 revealed a genome size slightly larger than those previously described for this genus, which typically ranges from 3.33 to 3.87 [44, 45]. The increased chromosomal size of *C. maltaromaticum* CM22 may contribute to its ability to colonize multiple and diverse habitats compared to other carnobacterial species [46].

**Fig. 4:**
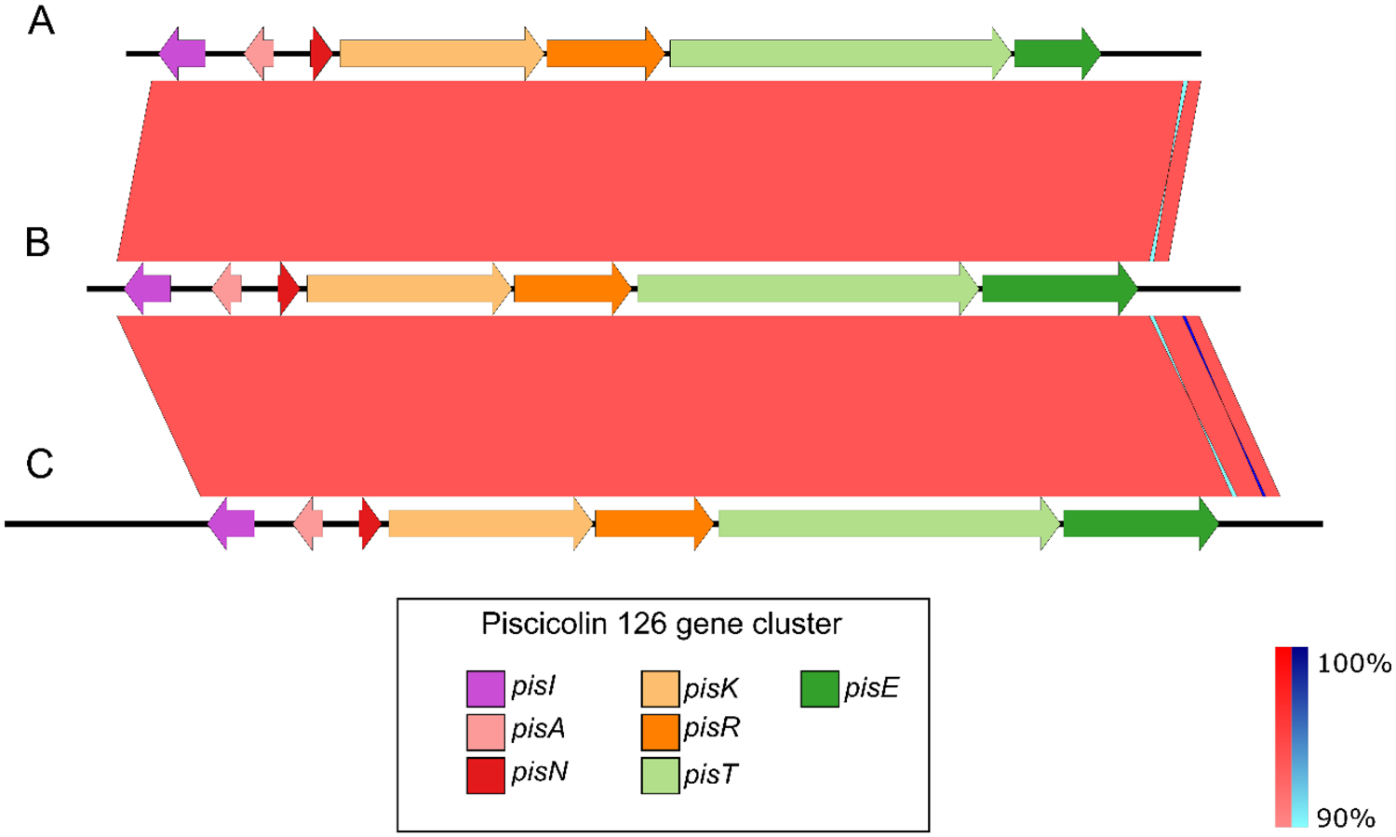
Homology between piscicolin 126 gene cluster of **A** *Carnobacterium maltaromaticum* UAL26, **B** *C. maltaromaticum* CM22, **C** *Carnobacterium piscicola* JG126. The color scale represents the similarity between the different genes: light red and blue = 90% similarity, while dark red and blue indicate up to 100% similarity; red represents for matches in the same direction and blue for inverted matches. The entire region of the cluster shows similarity in all three strains

Gursky et al. [42] described and analyzed the gene clusters responsible for the production of piscicolin 126 in the *C. maltaromaticum* UAL126 strain (accession number AY812745.1) and in the *C. piscicola* JG126 strain (accession number AF275938.1), respectively. A comparison between the piscicolin gene clusters of *C. maltaromaticum* CM22, *C. maltaromaticum* UAL26, and *C. piscicola* JG126, using by blast alignment, revealed 93.36% and 93.17 identity at the DNA level (Fig. 4). The gene cluster was arranged in the same direction for each of the ORFs identified. Individually, each protein of the gene cluster showed high similarity (> 85%) (Table 3).

**Table 3:** Comparison between *Carnobacterium maltaromaticum* CM22, *C. maltaromaticum* UAL26, and *C. piscicola* JG126 gene clusters. The table represents the similarity observed between the proteins of the clusters

***C. piscicola* JG126**	**Percentage identity (%)**	***C. maltaromaticum* CM22**	**Percentage identity (%)**	***C. maltaromaticum* UAL26**
PisI	96.94	PisI	95.92	PisI
PisA	87.10	PisA	87.10	PisA
PisN	85.11	PisN	85.11	PisN
PisK	90.19	PisK	90.19	PisK
PisR	96.73	PisR	96.73	PisR
PisT	96.37	PisT	95.95	PisT
PisE	94.48	PisE	91.67	PisE

## Discussion

Bacteriocins synthesized by LABs have gained significant attention for their potential applications as antibiotic alternatives and novel biopreservatives. Additionally, there is ongoing exploration into the potential use of these producer strains as probiotics [47]. In this work, we have isolated the *C. maltaromaticum* CM22 strain with a significant antimicrobial activity linked to the production capacity of piscicolin, which could suggest its potential technological use in the agri-food industry. The wide range of foods in which *C. maltaromaticum* has been identified renders this strain highly compelling for potential applications in the food industry. *Carnobacterium* strains have been mostly isolated from food, animal sources, and a wide range of environments [13]. Consistent with our results, other authors have reported the isolation of *Carnobacterium* strains from aquatic environments, including salmon [48] and extreme environments such as Antarctic ecosystems [49–52]. Some fish products have also been described as a source of isolation of *C. maltaromaticum* strains like vacuum-packed tuna [53], smoked surubim [22], and smoked salmon [54]. In addition, other authors have reported its isolation in meat products [55, 56].

Furthermore, in our investigation, we purified and characterized the inhibitory substance produced by *C. maltaromaticum* CM22, thereby confirming its identity as piscicolin CM22, a variant of piscicolin 126. Additionally, a genome study of the CM22 strain has been conducted. Gursky et al. [43] also analyzed the genome of the strains *C. maltaromaticum* JG126 and *C. maltaromaticum* UAL26 and observed that piscicolin 126 was encoded by an operon that included genes cataloged as ABC transporters, genes responsible for immunity, coding for histidine kinase protein, along with other accessory genes. Those findings show significant homology with our genomic analysis of *C. maltaromaticum* CM22, where an analogue of the piscicolin 126 cluster is maintained. This cluster comprises genes coding for bacteriocin immunity (MILBGHNJ_00131), histidine kinase (MILBGHNJ_00128), and ABC transporters (MILBGHNJ_00126). The proximity found between the *pisA* gene that encoded piscicolin CM22 (131,370–131558 bp) and the gene responsible for bacteriocin immunity (131,814–132,110 bp) is noteworthy.

The mass of piscicolin 126 produced by *C. maltaromaticum* JG126, as determined by MALDI-TOF, is 4416.6 Da [43], compared to the 4428.1 Da (theoretical Mw for the user-entered sequence 4431.93) obtained for the same bacteriocin produced by *C. maltaromaticum* CM22. The percentage of identity between these two proteins is around 87%, which explains this small variation (Supplementary Fig. 5S). Similar to the majority of described bacteriocins, the hydrophobicity plot of piscicolin CM22 suggests its nature as a hydrophobic peptide, with approximately 34.1% of its amino acid residues being hydrophobic. Additionally, it exhibits a notable basic character, with a predicted isoelectric point (pI) of 9.19.

Also, the genomes of strains UAL26 and JG126 had structural genes for the bacteriocin BM1. This bacteriocin contains the characteristic YGNGV motif of the family of class II bacteriocins in the N-terminal region [42, 57, 58]. A congruent result was observed compared to *C. maltaromaticum* CM22, wherein the bacteriocin BM1 genes are also present; however, bacteriocin BM1 production could not be detected under the assayed conditions.

LAB strains often harbor the genetic components necessary to produce multiple bacteriocins that can synergistic or additively work together, resulting in a stronger inhibitory effect against susceptible strains when expressed concurrently [59]. Cintas et al. [60] showed that LABs frequently possess genetic elements within their genomes for the production of multiple bacteriocins, since they tend to act synergistically, leading to a more pronounced inhibitory effect. However, other authors have argued that certain bacteriocins may exhibit antagonistic effect [61]. These mutually exclusive alternatives open the door to future research, exploring the production conditions of the bacteriocins piscicolin CM22 and BM1 and evaluating the effect that one exerts over the other. The genetic organization of piscicolin 126 on gene cluster depicted in Fig. 3 has been reported to be conserved between *Carnobacterium* and other LAB species such as *Enterococcus* [62]. Nevertheless, piscicolin 126 and carnobacteriocin BM1 clusters are not always present together in the same strains. Quadri et al. [42] reported that *Carnobacterium piscicola* LV17B strain is another producer of carnobacteriocin BM1 that does not contain the piscicolin cluster. In conjunction with the study conducted by Leisner et al. [13], both works showed that the structure of carnobacteriocin BM1 gene cluster is highly similar to that found in the present work.

In another study by Quadri et al. [41], the carnobacteriocin B2 gene cluster includes a structural gene, *cbnB2*, and a gene coding for bacteriocin immunity, *cbi*B2, providing autoimmunity not only to its own carnobacteriocin B2 but also to some other antimicrobials. These findings exhibit homology with the gene cluster of the carnobacteriocin BM1, where a structural gene (MILBGHNJ_02530) and a coding gene for immunity (MILBGHNJ_02531) are present.

In our work, we demonstrated the antimicrobial activity of *C. maltaromaticum* CM22 associated with its piscicolin CM22, revealing a broad spectrum of action in both in solid and liquid media against several Gram-positive bacteria relevant to human and animal health [63]. Given its antibacterial potential against both pathogenic and probiotic microorganisms, it is imperative to study and monitor the impact of these bacteriocin-producing strains on the gut microbiota. Preliminary studies, such as gut simulation trials on the target species, could be conducted [64, 65]. In addition to its antibacterial properties, this strain exhibits the capability to grow at 5 °C. This, along with its ability to grow at low temperatures, could facilitate its potential application as bioprotective agent for refrigerated food, inhibiting the growth of psychrotolerant pathogenic bacteria such as *L. monocytogenes* [22, 56] or other spoilage bacteria [44, 66]. Furthermore, *C. maltaromaticum* is recognized as a generally recognized as safe (GRAS) bacterium in the United States and it is considered a microorganism with beneficial technological use in the EU [67]. Although commercial products with this bacterium such as Micocin®, formed by a culture of the *C. maltaromaticum* CB1 strain, are already available for the preservation of meat products [12], *C. maltaromaticum* is not yet included in the European qualified presumption of safety (QPS) list due to some strains being associated with diseases in fish [68–70]. In contrast, some authors have highlighted the probiotic potential of *Carnobacterium* in different fish species, such as rainbow trout (*Oncorhynchus mykiss*) [52] and salmon (*S. salar*) [71]. For example, some authors have described the benefits of using *Carnobacterium* strains as probiotics in the diet of rainbow trout, exhibiting the ability to control pathogens such as *Aeromonas salmonicida* or to improve the immune response of fish [52, 72]. Recently, Puvanendran et al. [18] reported the ability of a strain of *Carnobacterium divergens* to increase growth parameters and disease resistance in cod larvae (*Gadus morhua*). In the present work, *C. maltaromaticum* CM22 demonstrated antimicrobial activity against a fish pathogen such as *Streptococcus phocae* subsp. *salmonis* [73]. Therefore, its potential use as a probiotic in the aquaculture sector is a promising avenue for research.

## Conclusion

In conclusion, our work has unveiled a novel psychrotolerant *C. maltaromaticum* CM22 strain, isolated from *S. salar*, capable of producing the bacteriocin piscicolin CM22. Further studies are needed to investigate its safety properties and potential applications in the food preservation sector, either as bioprotective culture for refrigerated food or through the applications of its purified bacteriocin. Additionally, its probiotic properties could be explored for potential applications as an active ingredient in feed production.

## Supplementary Information

Below is the link to the electronic supplementary material.

Supplementary file1 (PDF 356 KB)

## Data Availability

Bioproject PRJNA1004195 included the assembled genome of *C. maltaromaticum* CM22 deposited at NCBI under accession number JAVBVO00000000.1.
