# Supplementary material for: Genomic Characterization of Piscicolin CM22 Produced by Carnobacterium maltaromaticum CM22 Strain Isolated from Salmon (Salmo salar)
Source: Probiotics Antimicrob Proteins. 2024 Jul 3;17(6):3826–38. doi: 10.1007/s12602-024-10316-1 (PMC12634766; doi:10.1007/s12602-024-10316-1)
Supplement: Supplementary file 1 — Supplementary file1 (PDF 356 KB) [file 12602_2024_10316_MOESM1_ESM.pdf]

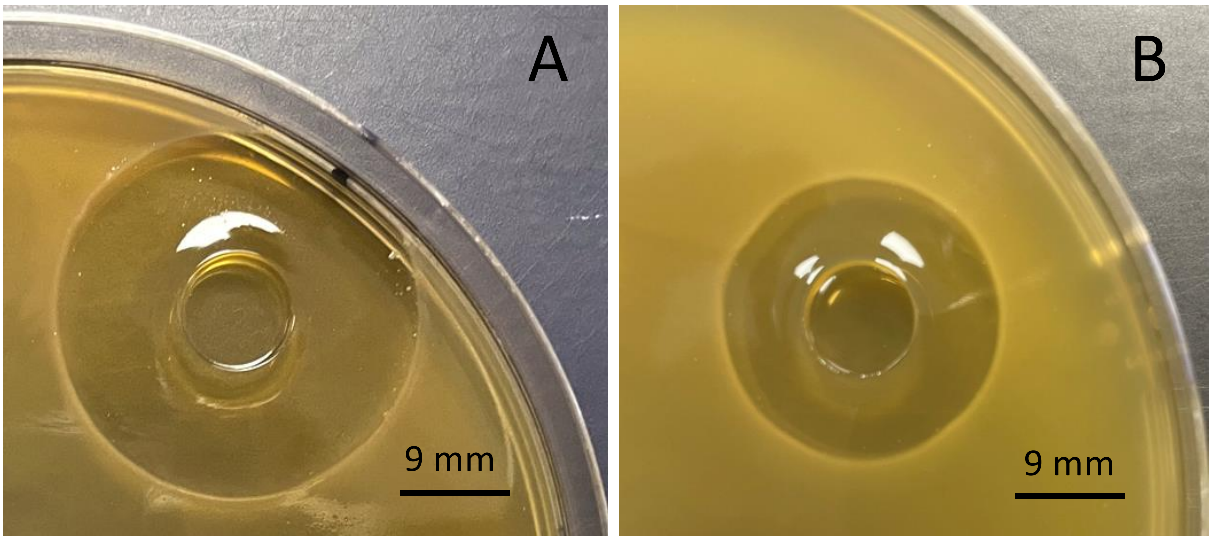


**Figure 1S**. Photograph illustrating the inhibition caused by *Carnobacterium maltaromaticum* CM22 culture supernatants against A) *Listeria monocytogenes* CECT4032 B) *Enterococcus faecalis* S-47.

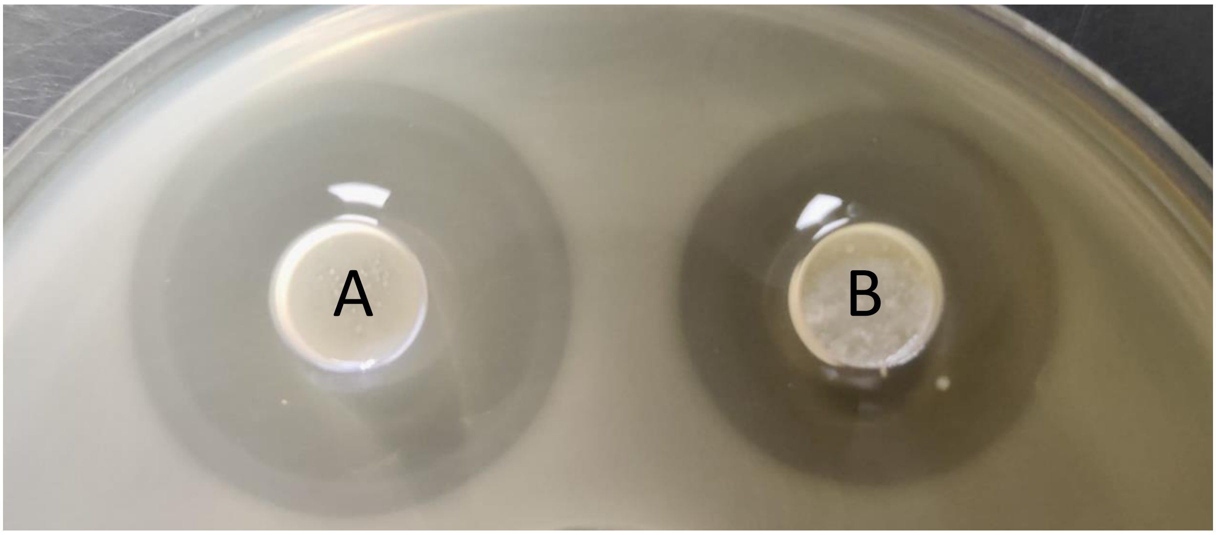


**Figure 2S**. Photograph illustrating the inhibition caused by cultures of A) *C. maltaromaticum* CM22 and *Pediococcus acidilactici* ST6 (García-López *et al,* 2023), a Pediocin PA-1 producer, against *Listeria monocytogenes* CECT4032.

García-López JD, Teso-Pérez C, Martín-Platero AM, Peralta-Sánchez JM, Fonollá-Joya J, Martínez-Bueno M, Baños A (2023) *Lactiplantibacillus paraplantarum* BPF2 and *Pediococcus acidilactici* ST6, Two Bacteriocinogenic Isolated Strains from Andalusian Spontaneous Fermented Sausages. Foods. 12(13): 2445. doi: 10.3390/foods12132445.

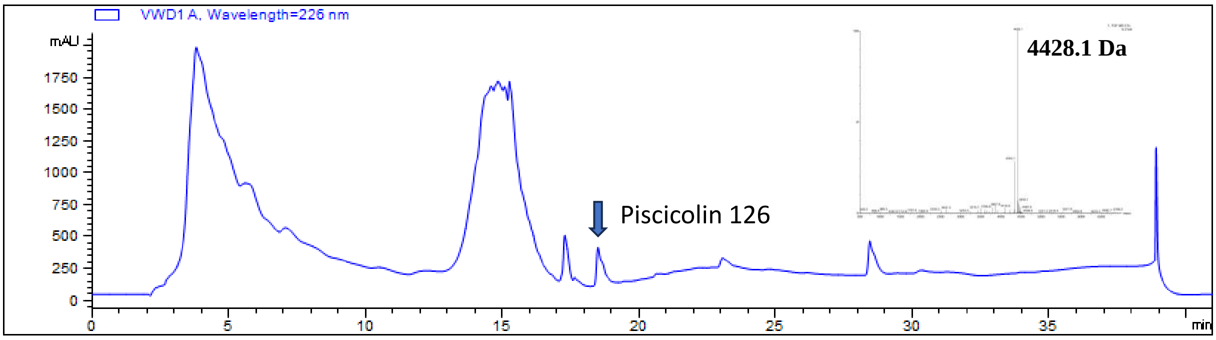


**Figure 3S.** Purification and detection of piscicolin CM22. Chromatogram of a semipurified fraction of *Carnobacterium maltaromaticum* CM22 purified by reversed-phase HPLC. The active fraction was analysed by mass-spectrometry (inset).

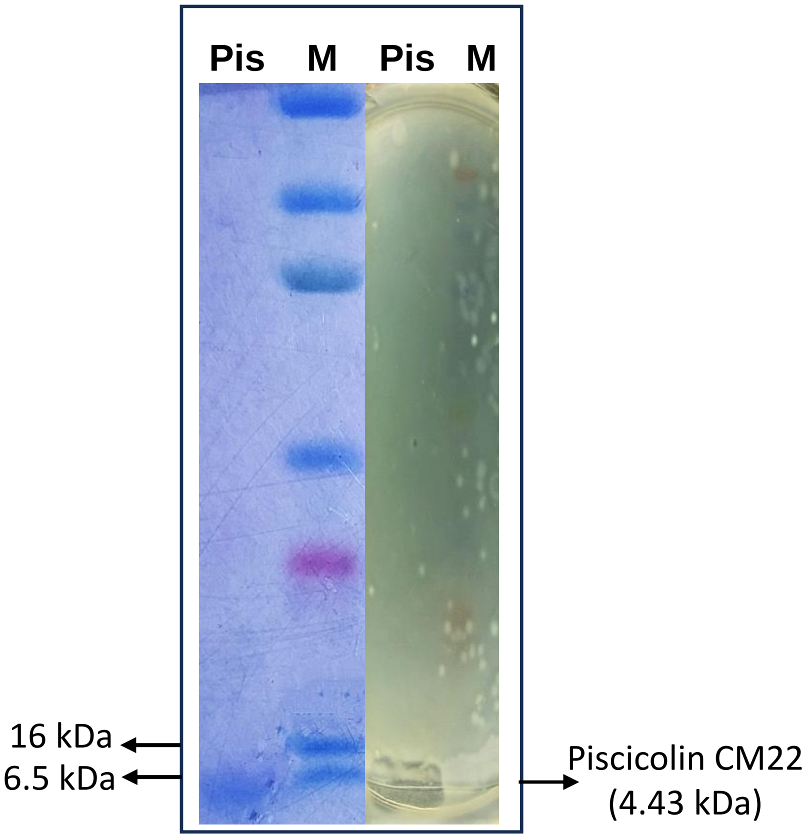


**Figure 4S.** SDS-PAGE analysis of semipurified piscicolin CM22 (fractions after reversed-phase chromatography). Left panel contains a Coomassie blue stained gel and on the right the gel was covered with soft agar inoculated with *Listeria innocua* CECT 4030 using the method of Bhunia et al. (1987). Pis: Piscicolin 126 sample, M: Nippon Genetics BlueEasy protein marker.

Bhunia AK, Johnson MC, and Ray B (1987) Direct detection of an antimicrobial peptide of *Pediococcus acidilactici* in sodium dodecyl sulfate-polyacrylamide gel electrophoresis. J Indian Microbiol 2: 319-322. doi: 10.1007/BF01569434

```
Pis 126 (UAL26)      KYYGNGVSCNKNGCTVDWSKAIGIIGNNAAANLTTGGAAGWNKG
Pis 126 (JG126)      KYYGNGVSCNKNGCTVDWSKAIGIIGNNAAANLTTGGAAGWNKG
Pis 126 (CM22)       KYYGNGLSCNKKGCTVDWGTAIGIIGNNAAANWATGGAAGWNKG
                     ******:****:******..************ :**********
```

**Figure 5S**. Sequence alignment of Piscicolin 126 (Pis 126) from *Carnobacterium maltaromaticum* UAL26, Piscicolin 126 from *C. maltaromaticum* JG126 and Piscicolin CM22 from *C. maltaromaticum* CM22.
